# The PDC framework applied to prosody and disfluency

**DOI:** 10.3389/fpsyg.2013.00232

**Published:** 2013-04-26

**Authors:** Fernanda Ferreira

**Affiliations:** Department of Psychology and Institute for Mind and Brain, University of South CarolinaColumbia, SC, USA

The Production-Distribution-Compre-hension (PDC) approach to language processing has the potential to be enormously productive because of its breadth and its elegant simplicity. As the name suggests, this framework relates phenomena from language production, typology, and comprehension in a specific direction: speakers' preferences determine the frequency with which linguistic forms occur; those frequencies determine form distributions; and frequent forms are predicted to be easier to comprehend, an idea that is based on classic and well-established principles of learning. Because the production system essentially leads this parade, the MacDonald article (MacDonald, 2013) focuses more on production than the other systems of language, which in many ways is a welcome change from the standard bias in psycholinguistics. Thus, in production, the approach neatly captures the sometimes competing forces that influence lexical and grammatical choices, which MacDonald (2013) refers to as Easy First, Reuse, and Reduce Interference. My goal in this commentary is to extend the PDC approach to two domains that are not discussed in the article: prosody and disfluency. These domains of language processing are obviously critically important for dialog and conversation, and they appear also to follow the basic principles of PDC, and in fact nicely illustrate its strengths.

As mentioned, MacDonald's analysis of language production assumes that speech is influenced by three preferences: Easy First, Reuse, and Reduce Interference (see article for details), which can be caricatured as Blurt, Mimic, and Space, respectively. Blurt is the tendency to initiate production with material that is more available or accessible in memory, Mimic is the tendency to repeat what you or your interlocutor has said, and Space is the tendency to separate difficult items using a range of devices including syntactic alternations, optional words such as *that*, and potentially also pauses, fillers (*um*, *er*), and syllable elongation.

In the realm of prosody and production, these three forces might lead to the following tendencies: First, Blurt encourages speakers to begin their utterances with the concept that is most semantically or phonologically ready (Levelt and Maassen, 1981; Bock, 1987), and to articulate accessible words in a way that requires minimal effort—that is, to make the words shorter, quieter, and generally less prominent. Blurt would thus lead to a tendency toward the so-called Given-New strategy, which states that speakers prefer to begin their utterances with information that has been established in the context, and to place novel contributions near the end (Haviland and Clark, 1974). Moreover, the Nuclear Stress Rule states that, in English, the default pattern is for main sentence stress to occur at the end of the utterance (Chomsky and Halle, 1968). The combination of Given-New and the NSR leads to sentences in which given elements tend to be early and less prosodically prominent, and new elements tend to be late and accented.

Mimic predicts that prosodic patterns can be primed just like syntactic structures. This idea has not received a great deal of attention in the literature thus far, but certainly some degree of prosodic repetition seems to be at work in conversation. To see how these two forces might influence prosodic forms, consider the question: *Who frightens Bill*? The Given-New strategy, now reformulated as a version of Blurt or Easy First, predicts that *Bill is frightened by everybody* would be a felicitous reply, and intuition suggests that it is. But if Mimic (Reuse) is more dominant than Blurt, the second speaker might be pushed into replying with *Everybody frightens Bill*—that is, to reuse the first speaker's syntactic and prosodic forms, the second speaker might also choose to create an active sentence, which now puts the easier material late in the sentence—contrary to Given-New. It's particularly interesting that these two strategies have different consequences for the prosody of the overall sentence. If Blurt wins, then the new information *everybody* is prosodically even more prominent than what is mandated by the NSR (*Bill is frightened by EVERYBODY*). But if Mimic wins, then the new information will get an early position in the utterance, but it also must be prosodically prominent (EVERYBODY frightens Bill). Thus, it can be argued that Blurt or Easy First pushes speakers to conform to the Given-New strategy, whereas Mimic or Reuse pushes them to use prosodic devices such as contrastive prominence. However, some languages, including Italian and other Romance languages, do not allow prosodic prominence early in utterances, as in *EVERYBODY frightens Bill*; syntactic devices for varying constituent prominence must be used instead (Samek-Lodovici, 2006). These languages would seem to somehow have developed grammatical constraints that favor efficiency related to lexical-phonological availability over efficiency related to pattern reuse. Finally, the third force, Reduce Interference or Space, is also potentially applicable to prosodic patterns in language. Space predicts that phrase-final lengthening and pausing will be used to separate elements that are potentially interfering, which seems consistent with findings in the literature (e.g., Smith and Wheeldon, 2004) and is assumed in some models of language production and prosody (Watson and Gibson, 2004).

Disfluencies are a closely related topic of increasing interest in the field of psycholinguistics (e.g., Arnold et al., 2003; Ferreira et al., 2004). The PDC framework offers a useful way to think about the relationship between disfluency production, distribution, and comprehension. Again, in production, three forces are at work: Blurt, Mimic, and Space. Put very simply, it seems plausible that when Blurt and Mimic dominate, speakers will tend to generate sequences that will occasionally need to be repaired; and when Space dominates, people might tend to produce “pauses” of various sorts, including not just silent intervals but also *uh*s, *um*s, and repetitions (*the the aardvark*). If a speaker begins an utterance with a concept that is highly available—that is, if she blurts—she might find herself producing an infelicitous sequence, or possibly even an opening that appears unlikely to have a grammatical outcome (e.g., if the object of a preposition is the most active concept and the speaker begins an utterance with it, a repair may be required, as in *the trunk—uh put the beer in the trunk*). This tendency has been observed in our studies of language production focusing on individuals diagnosed with Attention Deficit Hyperactivity Disorder (ADHD) (Engelhardt et al., 2009): People with ADHD who were required to generate an utterance with a past participle form such as *ridden* were more likely to begin their utterances with an animate entity (*the girl ridden*) which then necessitated a repair (*the girl—uh the bicycle was ridden by the girl*) than were age- and demographically matched subjects with no history of ADHD. Given that ADHD is associated with problems relating to inhibitory control, it might be expected that individuals suffering from it would be more likely to follow Blurt or Easy First, often triggering repairs. Mimic or Reuse should work similarly—given that simply mimicking another person's utterance will sometimes impede communicative success, we can predict that individuals in whom this force dominates over Blurt or Space will tend to produce sequences that are infelicitous or just plain wrong, leading to the need for repair. This idea has not been tested yet, to the best of my knowledge, but the logic seems fairly straightforward.

Reduce Interference, as mentioned, leads speakers to space apart difficult elements, and therefore might lead speakers to produce *uhs*, *ums*, and repeats. Indeed there is evidence that concepts that are hard to retrieve are sometimes preceded by filler disfluencies (Arnold et al., 2003). Perhaps more interesting are cases in which speakers appear to take advantage of lexical and syntactic alternatives to buy themselves extra planning time during production, in effect treating these linguistic elements as disfluencies. As MacDonald notes in her article, speakers more often include the optional *that* in sentence complement structures when the first phrase of the embedded clause is difficult to retrieve. One especially compelling demonstration of this tendency comes from Ferreira and Firato (2002), who used a sentence recall paradigm to induce speakers to produce sentences with conceptually similar noun phrases, as in *The author, the poet, and the biographer recognized (that) the writer/golfer was boring*. They observed that speakers were more likely to include the optional *that* and to produce disfluencies when the subject of the embedded clause was *writer* rather than *golfer*. This effect was observed because interference builds up over the matrix subject, and so the speaker takes advantage of the optional *that* to space the difficult-to-retrieve item from the ones that preceded it. Recently, the Uniform Density Hypothesis has been offered to capture speakers' tendency to distribute information evenly across an utterance, which in part explains the inclusion or omission of optional grammatical words such as complementizers (Jaeger, 2010). The PDC framework assumes that Uniform Density is a product of Reduce Interference or Space, and so is only one force at work during production. Indeed, it would be expected that when Blurt or Mimic dominate over Space, information would not be uniformly distributed over the utterance.

This framework can also be turned around to help explain how linguistic devices encourage fluency. Blurt allows the speaker to begin an utterance without the need for *uh*s and u*m*s, because the speaker can start with what is already available. Utterance priming associated with Reuse or Mimic allows the speaker to focus on lexical choices rather than syntactic or prosodic planning, and promotes alignment between interlocutors (Pickering and Garrod, 2004). And Space would allow the use of prosodic, syntactic, and lexical devices to separate potentially interfering linguistic elements without the need for repeats or *uh*s and *um*s. Fluent production, in other words, requires that the three forces MacDonald has identified be optimally balanced. Conversely, disfluencies will occur when one of the forces is out of balance; depending on which one that is, different types of disfluency are predicted to occur.

The PDC framework thus appears to be a powerful approach for understanding language processing. Its distinctive contribution is to unite production and comprehension in a highly specific and testable way, and I hope that this piece demonstrates the extent to which it may be applied to phenomena not explicitly discussed in the article. Of course, many outstanding questions and issues remain. For example, it is not clear how this approach to linking the production and comprehension systems differs from that of Pickering and Garrod (2004), who appear to have different but not incompatible goals: whereas MacDonald would like to explain how distributional patterns arise which then influence comprehension, Pickering and Garrod's goal is to describe how the two systems work together in real time to facilitate conversation and dialog. It goes without saying that such a treatment is well beyond the scope of the MacDonald article (MacDonald, 2013), but eventually it would be useful to reconcile or at least compare and contrast these different frameworks, given the similar aims of both. It also would be helpful to see more examples of linguistic ambiguities that the PDC approach can explain. But at this point it is enormously helpful to have a clear, coherent framework from which to ask these questions and to conduct future empirical investigations.
